# “There is No Reliable Evidence to Pass Moral Judgment on Frauwallner.” Erich Frauwallner, Jakob Stuchlik, Walter Slaje, and the Whitewashing of Austrian Indology During the Time of National Socialism

**DOI:** 10.1007/s00048-023-00366-x

**Published:** 2023-09-06

**Authors:** Eli Franco

**Affiliations:** Universität Leipzig, Leipzig, Germany

**Keywords:** Erich Frauwallner, Jakob Stuchlik, Walter Slaje, Indology and National Socialism, Erich Frauwallner, Jakob Stuchlik, Walter Slaje, Indologie und Nationalsozialismus

## Abstract

This paper engages with a little-known controversy between Jakob Stuchlik and Walter Slaje on the involvement of Erich Frauwallner, the renowned scholar of Indian philosophy (1898–1974), with NS institutions. It sheds new light on this controversy and highlights the Aryan-supremacist ideology that is reflected in Frauwallner’s division of the history of Indian philosophy into an Aryan and non-Aryan period. On the whole, the paper sides with Stuchlik and exposes Slaje’s attempt to whitewash Frauwallner and certain aspects of his work, despite his adoption of NS ideology and involvement with NS institutions such as the Gestapo and SA. Moreover, the paper dwells on Frauwallner’s adherence to antisemitism and Aryan-supremacist ideology even after the WWII and as late as the 1960s.

## Abstract (de)

Dieser Beitrag befasst sich mit einer wenig bekannten Kontroverse zwischen Jakob Stuchlik und Walter Slaje über die Verstrickung von Erich Frauwallner, dem renommierten Gelehrten der indischen Philosophie (1898–1974), mit NS-Institutionen. Er wirft ein neues Licht auf diese Kontroverse und zeigt die arisch-supremistische Ideologie, die sich in Frauwallners Aufteilung der Geschichte der indischen Philosophie in eine arische und eine nichtarische Periode widerspiegelt. Im Großen und Ganzen stellt sich der Aufsatz auf die Seite von Stuchlik und entlarvt Slajes Versuch, Frauwallner und bestimmte Aspekte seines Werkes zu beschönigen, obwohl er sich die NS-Ideologie zu eigen machte und mit NS-Institutionen wie der Gestapo und der SA zusammenarbeitete. Darüber hinaus befasst sich der Beitrag mit Frauwallners Festhalten an Antisemitismus und arisch-supremistischer Ideologie auch nach dem Zweiten Weltkrieg und noch in den 1960er Jahren.

The history of German Indology during the dark time of National Socialism remains to be written. So far, some 75 years after World War II, except for studies about the notorious cases of Jakob Wilhelm Hauer (see Junginger 1999; Ulrich Hufnagel 2003; and Junginger 2003), a scholar of religion, and Walther Wüst (see Junginger 2008 and numerous further references therein), an Indo-Europeanist, both with a focus on India, and Jakob Stuchlik’s 2009 monograph on Erich Frauwallner discussed here, only a handful of papers on the subject have been published, notably the programmatic paper by Sheldon Pollock, “Deep Orientalism? Notes on Sanskrit and Power Beyond the Raj (Pollock 1993)”.^1^ If I am not mistaken, there are two remarkable facts about studies of Indology at the time of National Socialism: one, no study of German Indology during this time was undertaken before 1993; two, no study of German Indology during this time was undertaken by any German Indologist up to the present day.^2^ (I use the term ‘German Indologist’ for Indologists employed at German universities or academic institutions irrespective of their nationality.) The contribution of German Indologists to this subject has thus far been mainly negative, in the form of objections to the validity of the very few studies undertaken by others.^3^ Pollock’s paper has been criticized, partly with superfluous ad hominem arguments by Grünendahl (2006 and 2012). Stuchlik’s study has mainly been criticized by Walter Slaje in a lengthy review (Slaje 2010), which will be discussed here in some detail.^4^

Jakob Stuchlik’s analysis of Erich Frauwallner’s entanglements is an extensive study of a prominent Indologist whose work dominated the field of the history of Indian philosophy throughout the second half of the twentieth century—at least in the German-speaking world—and is still of scholarly relevance even today, but who was also an antisemite, a staunch supporter of the National Socialist German Workers’ Party (NSDAP), and a racist who never seems to have doubted the supremacy of the Aryan race and, specifically, of its Germanic branch. Furthermore, Stuchlik’s study is an important part of the Austrian Academy of Sciences’ attempt to come to terms with its own past (Steinkellner 2009). Some fourteen years after its publication, one must note that it has neither received the response it deserves nor led to further studies. On the contrary, after the extremely vicious attack on Stuchlik by Walter Slaje (and earlier on Pollock by Reinhold Grünendahl), I doubt that any German Indologist will be inclined to engage with the history of their discipline during the National Socialist (NS) era for the foreseeable future. It is therefore with high hopes that I have followed the establishment and unfolding of a project on “Indologie im nationalsozialistischen Deutschland,” funded by the German Research Council (DFG), directed by Moritz Epple in collaboration with Diya Roy, and conducted with a carefully considered methodology and a multidisciplinary approach. This project, initiated in 2018, is bound to deepen our understanding and provide a firm and reliable foundation for studying the ways Indologists interacted with political institutions in the NS era. The following paper is written in support of this project.

The following pages narrate a remarkable, yet little known, controversy^5^ concerning the study of Indology in the NS era. Its focus, however, as the title of this article indicates, has as much to do with the present, namely with the blatant attempt by Walter Slaje, an emeritus professor of Indology at Halle University, to whitewash Erich Frauwallner and certain aspects of his work, despite his adoption of NS ideology and deep involvement with NS institutions. I will first briefly present the results of Stuchlik’s work, with additional comments and observations, and then explain why Slaje’s treatment of this scholarship is disgraceful. Whatever misgivings one may have about Stuchlik’s study, it should not be rejected, or worse—for this is obviously the purpose of Slaje’s review—simply ignored. What Slaje would have us believe is that the book has neither merit nor value, that it is irrelevant insofar as the NS period is a long-overcome thing of the past, that Frauwallner was not a racist, and, in short, that the book should not have been published, at least not by a reputable publisher such as the Austrian Academy of Sciences Press. However, it is difficult to imagine that, after having studied Stuchlik’s book, an unbiased reader would concur with Slaje’s conviction that Frauwallner’s behavior and beliefs were morally above reproach, or, as Slaje boldly asserts, that “there is no reliable evidence to pass moral judgment on Frauwallner (Slaje 2010: 453).”^6^

## Frauwallner’s Beginnings^7^

Erich Frauwallner, son of the civil servant Dr. Friedrich Georg Frauwallner and his wife Maria Barbara née Riedler, was born in Vienna on December 28, 1898. He received his secondary education first at the State High School (Staatliches Gymnasium) in the 7th district of Vienna, and later on at the Academic High School (Akademisches Gymnasium). Drafted into the Imperial Army on May 11, 1916, he was granted special graduation while on leave from military service (Kriegsmatura) on November 17, 1916. During WWI, he took part in the “Battle of Isonzo” and the Romanian offensive, and then returned to Italy to serve in Udine. He was officially released from military service at the end of the war on November 30, 1918, though he must have reached Vienna slightly earlier, for he was already enrolled at the University of Vienna in the winter term of 1918/1919. As soon as he entered university, he joined the pan-Germanic and antisemitic association “Deutscher Turnerbund 1918” (sic, probably a typo for 1919) and the “Vandalia” fraternity (Burschenschaft).

Frauwallner studied classical philology, supplemented by courses in Indian and Iranian studies. After six terms, one of which he spent in Sweden at the University of Göteborg (Gothenburg), he submitted a doctoral thesis in classical philology entitled “De synonymorum quibus animi motus significantur, uso tragico” [“On the synonyms by which the movements of the soul are signified, in tragic usage”] on June 30, 1921, in which he examined the notions of wrath (*ira*), hate (*odium*) and suffering/pain (*dolor*) in the works of Aeschylus, Sophocles and Euripides; he took the so-called rigorous examinations (strenge Prüfungen) in July 1921 and subsequently obtained his doctoral degree. Having studied for a further year, he took the state exam for high school teachers (Lehramtsprüfung) on June 17, 1922, and took up a position as teacher of Classical Greek and Latin in a junior high school (Mittelschule) in Vienna’s 20th district.

Frauwallner’s first attempt (1926) to gain the *venia legendi* for Indology failed, but he was encouraged to apply again when he could present a more substantial volume of publications. He was eventually awarded the *venia legendi* on April 16, 1928, but took up teaching at the University, without remuneration, on a sessional basis (Lehrauftrag) only in the winter term of 1930/1931. The Ministry of Education turned down an application submitted by the Faculty of Philosophy for special funds to remunerate him, likely for political reasons, but reduced Frauwallner’s teaching load at the secondary school from seventeen to ten hours per week. During the 1930s, Frauwallner established himself as one of the leading scholars on the Buddhist epistemological tradition, publishing important papers practically every year on topics such as Buddhist philosophy of language (*apoha*), the notion of momentariness (*kṣaṇikatva*) in Buddhist philosophy, early Buddhist manuals of debate, and valid relations in Buddhist inferences and syllogisms. The special scholarly tradition founded by him, with which I am also partly affiliated, continues to be fostered in Vienna to the present day; academically speaking, it is now in its fourth generation.

## Working for the Party

As early as the end of the 1920s, Frauwallner must have already been known to the police as an ardent supporter of National Socialism. Stuchlik quotes a confidential police report from 1935, but referring to the time before 1933, which describes Frauwallner “as an avid attendee of National Socialist gatherings, […] who, according to reports, also spoke at such a gathering himself” (Stuchlik 2009: 123).^8^ He joined the NSDAP early, on November 29, 1932. In June 1933, when the Party became illegal in Austria, Frauwallner began working for the intelligence service of the Party (Vienna district), the SA and the Gestapo (more on that below). In December 1933, six months after the NSDAP was outlawed in Austria, the Austrian bishops signed an epistle called the Weihnachtshirtenbrief. It contains a clear condemnation of the NSDAP (Stuchlik 2009: 107–108). Stuchlik quotes extensively from this letter; here are just a few sentences:

No human being is naturally worthier or loftier than another human being. […] Bombs and grenades, heavy duty firecrackers and explosives are not allowed under civil law, but only under martial law. […]

Humankind is a unitary family, based on justice and love. That is why we condemn the National Socialist racial madness, which leads to, indeed must lead to, racial hatred and conflicts between nations; we also condemn the unchristian law of sterilization. […]

That is why we preach the virtue of Christian patriotism, condemn the betrayal of the fatherland and condemn the radical racial antisemitism. […]

That is why we condemn the extreme nationality principle, defend the historical rights of our fatherland and welcome the cultivation of the idea of Austria.

This was most commendable, one would think. Frauwallner, however, found this epistle so objectionable and offensive that he decided—according to the police report quoted by Stuchlik—to organize a movement of mass-withdrawal from the Church in response. A few weeks later, the police were at his door. His apartment was searched. Letters related to the subject were found, but Frauwallner declared that they concerned only his individual intention to leave the Church and that he did not intend to promote apostasy (“Abfallspropaganda”). The policemen were hardly convinced. He remained under suspicion, but no further action was taken against him (Stuchlik 2009: 107–108).

The relationship between the NSDAP and the Catholic Church seems to have been Frauwallner’s special field of interest and activity for the Party. The Church was a force to be reckoned with in deeply Catholic and conservative Austria, and enlisting its support for the NS regime in the initial stages of the German annexation of Austria (the so-called Anschluss) was crucial. According to his own words, Frauwallner worked for the Party, the SA and the Gestapo intelligence services by providing information “on the state of things in the area of religion and the effective forces therein” (Stuchlik 2009: 108). Frauwallner also participated in the “Working Group for Religious Peace” (Arbeitsgemeinschaft für religiösen Frieden), obviously representing the interests of the Party, not the Church (Stuchlik 2009: 91). The purpose of this Arbeitsgemeinschaft, which was founded in the early 1930s, was to work out common ground between the Church and the NSDAP in order to enlist the support of the former for the latter. In a letter by its secretary Johann Pircher, dated April 26, 1937, Frauwallner was considered to be (Stuchlik 2009: 110) “possibly the oldest active spearhead of the idea of establishing peace in terms of religion and worldview”. At some point, no later than 1938 (Stuchlik 2009: 91), Frauwallner’s role in the Arbeitsgemeinschaft für religiösen Frieden was upgraded, and he became the deputy (Stellvertreter) of Karl Pischtiak, another “old fighter” who was most probably involved in the July Putsch, the personal adviser of Joseph Bürckel (one of the highest ranking NSDAP members in Austria), and Bürckel’s point man in Church matters. Frauwallner’s exact role in this context can no longer be reconstructed, but it must have been considerable, as is clear from a diary remark by Pischtiak dated March 11, 1938, the day of the Anschluss, according to which he, his deputy Frauwallner and Anton Böhm, the editor of the Catholic weekly *Schönere Zukunft*, discussed “an action plan” (Stuchlik 2009: 111).

On May 27, 1938, Frauwallner declared in his application for renewal of his party membership card that he was a member inter alia of the right-wing Austro-Fascist “Vaterländische Front”, the “Burschenschaft Vandalia”, and the “Deutscher Schulverein—Südmark”. Further, under the heading of “activity for the NSDAP” after the prohibition of the NSDAP in Austria on June 19, 1933, he mentioned that had been “working for the intelligence service of the Vienna district, the Vienna group of the SA, and the Gestapo (Dr. Begus); most recently for the working group concerning national politics (Stuchlik 2009: 35, n. 15).” Yet after WWII, in a statement made on January 2, 1947, at the police headquarters at Fieberbrunn (Tyrol), Frauwallner essentially denied that this had been the case. Contrary to his 1938 application, he claimed that he had mentioned “some unimportant matters” (“einige Nichtigkeiten”) to further his case, referring in particular to his statements on working for the intelligence service of the Party, the SA and the Gestapo. He thus implied that he was actually just pretending at the time: “Through a precise investigation, it will definitely be possible to determine that I am not known in any of the institutions named in the application, such as the intelligence service of the district of Vienna or the SA group” (Stuchlik 2009: 89).

Did Frauwallner really lie so boldly to the NS authorities in 1938? Or did he instead lie to the post-WWII authorities in Austria in 1947? As is obvious from Stuchlik’s investigation, Frauwallner was not literally lying, but he was certainly trying to deceive the new Austrian authorities. As it turned out, Frauwallner did not report directly to the SA and the Gestapo, but through a go-between. In a statement made on April 2, 1947 (three months later) to the district attorney’s office (Staatsanwaltschaft) in Vienna, Frauwallner admitted that during the time when the NSDAP was illegal, he was providing information “on the state of things in the area of religion and the effective forces therein” (Stuchlik 2009: 90) to a person by the name of Robert whose surname Frauwallner could no longer remember and who was connected to various intelligence services.

Who were these two individuals named by Frauwallner—“Dr. Begus” and “Robert”? As highlighted by Stuchlik, Dr. Otto Begus was a police superintendent in Vienna who is today remembered for his most probable participation in the assassination of the Austrian Chancellor Engelbert Dollfuß in 1934. He was also a member of the illegal NSDAP and the SS. In 1933, he was suspended from police service and sentenced to six months in prison for passing on sensitive information to the NS intelligence service. After serving his sentence, Begus escaped across the border to Germany in 1934 and became the head of the Munich-based Austrian Department of the intelligence service of the SS. In the summer of 1934, he returned to Austria, apparently in order to plan the July Putsch and the assassination of Dollfuß. He was arrested on August 11, 1934, for illegal activities and sentenced again to six months in detention. Released in February 1935, Begus returned to Germany and was transferred to Berlin to lead the “Büro T.G. des Forschungsamtes im Reichsluftfahrtministerium und des SS‑S.D.”, where he worked for a few months; he then participated in the Italian–Abyssinian War on the Abyssinian side (Stuchlik 2009: 98). As documented by Stuchlik, in 1936, Begus became a detective superintendent in the Frankfurt police headquarters, where he still served when, in May 1938, Frauwallner used his name as a possible witness to his party activities during the period when it was illegal. In June, Begus was transferred to Salzburg. Once the war began, he moved to the Waffen-SS, worked for the intelligence services in occupied France and Greece, and perhaps also in Theresienstadt (Stuchlik 2009: 101). After the war, Begus was convicted of war crimes and sentenced to three years of severe detention (schwerer Kerker); all his assets were confiscated (Stuchlik 2009: 114, n. 573). This is the man Frauwallner claimed to have collaborated with and worked for when the NSDAP was illegal in Austria.

As for the mysterious “Robert”, Stuchlik argues that this was most probably Robert Meissl, who was also arrested several times in connection with the Begus affairs (Stuchlik 2009: 97, 103). In 1934, he escaped to Munich and also worked for the LLÖ (Landesleitung Österreich). He worked in the courier service, smuggled weapons across the border, and operated as a political reporter. Together with Begus, he returned to Austria ahead of the July Putsch, although unlike Begus he does not seem to have been connected to the Dollfuß assassination. In 1935, Meissl became SS-Oberscharführer, and in 1936 “Geschäftstellenleiter für den Obersten Ehren- und Disziplinarhof der Deutschen Arbeiterfront” (Stuchlik 2009: 101). After the Anschluss, he returned to Vienna and probably worked for the Ministry of Economics and Labor. Such were the people Frauwallner chose to associate himself with. And yet, Slaje is unable to see any reliable evidence with which to pass moral judgment on Frauwallner. In fact, Slaje has decided that Frauwallner’s involvement with the intelligence services of the NSDAP, the SA, and the Gestapo is not even worth mentioning.

## The Takeover of Jewish Positions

Only one week after the Anschluss, on March 18, 1938, Frauwallner was transferred to the University (Stuchlik 2009: 61). One day later, he was formally appointed as a private university reader (Privatdozent) by Adolf Hitler and sworn in by him (not personally, of course). Here, one wonders whether the occupying Germans did not have more important things to do in their first week in Austria than immediately busy themselves with promoting “a public nobody” like Frauwallner, as Slaje calls him. In any case, Frauwallner first replaced the librarian at the Department of Oriental Studies, the renowned Jewish Assyriologist Leo Oppenheim, who was relieved of his duties on racial grounds on March 23 (Stuchlik 2009: 61, n. 223). On April 23, Bernard Geiger, a scholar of Iranian and Indian philology, who had kept Sanskrit Studies alive at the Department of Oriental Studies after Leopold von Schroeder’s death in 1920, lost his job; on the same day, Frauwallner was entrusted by the Dean with the continuation of Geiger’s lectures. In August 1939, Frauwallner was finally appointed extraordinary professor for Indology and Iranian Studies (Stuchlik 2009: 63).

Stuchlik accuses Frauwallner of participating in the “theft” of Jewish positions (Stuchlik 2009: 130).^9^ To be sure, Frauwallner did not personally remove Oppenheim, Geiger or anyone else from their positions. In terms of morality, he can be compared not to a thief, but to someone who knowingly buys stolen goods. Slaje, on the other hand, sees nothing amiss here. He lists, with deliberate and tendentious imprecision, the facts that Stuchlik uncovered and then dismisses everything with a few sarcastic words. Here is how Slaje haughtily makes fun of Stuchlik’s research: “After the ‘Anschluss,’ almost half (45%) of all Professors at the University were “put into early retirement” (63) in Austria by a decree dated May 28, 1938.^10^ The provision also ‘applied to’ Bernhard Geiger ‘as a member of the Jewish people.’ One and a half years later, on August 31, 1939, Frauwallner was appointed. Job theft” (Slaje 2010: 454). In other words, according to Slaje, Frauwallner’s appointment as extraordinary professor for Indian and Iranian Studies (Indologie und Iranistik) had nothing to do with Geiger’s loss of that very position, and Stuchlik’s accusation that Frauwallner participated in the theft of Jewish positions is ridiculous. Frauwallner had done nothing wrong and “there is no reliable evidence to pass moral judgment on Frauwallner”.

Frauwallner actually had an opportunity to come clean when he wrote a paper on the history of Indology in Vienna in 1961. He chose, however, not to take up the issue. Instead, he arrogantly belittled Geiger’s scholarly accomplishments:

Little can be said about Geiger’s activities as the representative of Indology in Vienna. His scholarly publications during this time are limited to a few short articles. The Indological section of the Department of Oriental Studies looked very much the same when he left in 1938 as when Schröder died. Thus, eighteen years had gone by during which the discipline developed rapidly (1982b [1961]: 32).

Not a word is lost here on the reasons for Geiger’s “Ausscheiden”—the ambiguity of the German word used by Frauwallner, which can evoke retirement, resignation, among other things, is noteworthy. Frauwallner never acknowledged the removal of Jewish academics from the University and the promotion of NSDAP loyalists to their positions.^11^ Indeed, after WWII, Austrian universities made no effort to regain the Jewish academics who had lost their positions in 1938; the few who tried to return were made to feel unwelcome (see Fleck 1966; Ackerl & Schödl 2005; also Rathkolb 2013).

## Valerie Walter’s Flat Occupied by Frauwallner^12^

In August 1938, the Central Department for the Emigration of Jews in Vienna was established under the effective management of Adolf Eichmann. Its purpose was to expel Jews from Austria and confiscate their property. As reconstructed by Stuchlik, Leo Walter and his wife Valerie, together with a relative, left their two-flat home on the first floor of Sieveringerstraße 16 in Vienna’s 19th district on November 19, 1938, ten days after the bloody pogrom euphemistically called “Reichskristallnacht” at the time—as if only crystal as had been broken that night. A few days later, on December 12, 1938, Frauwallner and his family moved into the flat, which was located in the half of the building owned by Valerie Walter. He certainly did not pay any rent to the owner, whose whereabouts were unknown; the three former inhabitants of the flat were officially recorded only as “departed”. The half the building, together with its garden, that belonged to Valerie Walter was auctioned off by the state in June 1941. Prior to that, Frauwallner enlisted the support of the district committee of the NSDAP in order to gain permission to act as the sole bidder. “Contrary to expectations” permission was not granted, and the relevant part of the building was auctioned off and sold to another “Aryan”. To support his case, Frauwallner had affirmed not only that he and his family were Aryans in the sense of the Nuremberg laws, but also that “up until now I have not acquired any Jewish assets”.^13^ After the war, Valerie Walter applied for the restitution of her property, and in 1949 had to “buy” it back from the new owner for approximately double the price it had been acquired for at the auction; her claim for disbursement of the rental income generated by the property up to that time was rejected. The Frauwallner family continued to live in the flat until at least 1948.

On this chapter of Frauwallner’s life, which has been competently researched by Stuchlik on the basis of original documents in various archives, Slaje has only one sarcastic remark to make: “As if that was not enough [i.e., the attempt to appropriate positions held by Jewish scholars for himself, EF], Frauwallner ‘committed an offense’ even with regard to ‘Jewish real estate.’ (p. 130) There is no end to Frauwallner’s moral abyss” (Slaje 2010: 454). Suppose the Slaje family had to leave their country because of pogroms and persecution, and that their neighbor moved into their home and tried to acquire it for a fraction of its value—do you reckon that Professor Slaje would see here no reliable evidence with which to pass moral judgment?

## Frauwallner’s Publications During WWII and the “Aryan Component” in Indian Philosophy^14^

During the war years, Frauwallner did not publish much. In fact, for more than a decade, from 1939 until 1950, only two papers can be mentioned in addition to a few short book reviews, and both are ideologically tainted: “Der arische Anteil an der indischen Philosophie” (The Aryan Component of Indian Philosophy) in 1939 and “Die Bedeutung der indischen Philosophie” (The Significance of Indian Philosophy) in 1944. In “The Aryan Component of Indian Philosophy”, Frauwallner begins by noting the striking similarities between European and Indian philosophies, and claims that since there was no direct mutual dependence linking these traditions, these similarities can only be explained by the fact that both philosophies were created by nations of the same “blood”, namely, by Aryan nations. However, because Indian philosophy contains much that is foreign and peculiar (“fremdartig, absonderlich”), it has to be assumed that the cultural development of India was influenced by non-Aryan indigenous people (“Urbevölkerung”). The task of the scholar of Indian philosophy was thus to determine what is to be considered Aryan within Indian philosophy.

Frauwallner argues that the history of Indian philosophy can be divided into two periods. The first period begins in Vedic times, experiences a peak with the philosophical systems developed in the first half of the first millennium CE, and then—after a period of decline—ends toward the close of the first millennium CE. The second period begins with the philosopher Śaṅkara and continues until the eighteenth century, when the introduction of Western ideas under British dominion puts an end to the development of indigenous Indian philosophy. The transition from the first to the second period cannot be explained as a continuous, unitary development. Rather, a dramatic change took place. The older systems (namely the philosophies of Sāṃkhya, Vaiśeṣika, Lokāyata, Buddhism and Jainism) were atheistic in the sense that they did not rely on a supreme god as a basic principle. They were not, Frauwallner says, religiously and dogmatically bound, but strove to derive their teachings scientifically without presuppositions (1939: 271).^15^ The new systems were theistic, and the divine revelation by Śiva or Viṣṇu was acknowledged as the supreme source of knowledge. In view of the fact that the religions of Śiva and Viṣṇu are non-Aryan in origin, one has to explain this radical change in the nature of Indian philosophy as the victory of non-Aryan nature over the dwindling strength of the Aryan spirit that was creative in the older systems.^16^

In “The Significance of Indian Philosophy”, Frauwallner basically repeats the same periodization of Indian philosophy. The upshot of his presentation is that the importance of Indian philosophy lies not only in its richness, but also in the fact that it developed independently of the European tradition, and that Indian philosophy is the only non-European tradition that can be compared to the European philosophical tradition. Only these two traditions are comparable because they alone have a scientific character, which, for example, the Chinese tradition lacks (Frauwallner 1944: 162); this is manifest in the fact that epistemology and logic are at the basis of every proof and in the methodically precise way in which the principal tenets of the systems are derived and substantiated. Frauwallner claims that the old systems are atheistic and in a scientific manner free from presuppositions. According to him, these and other similarities of the Indian and European traditions can be explained by the same propensity (“Veranlagung”), which is racially conditioned. The principal transformation of the Indian people that brought about the formation of Hinduism was due to racial reasons and can be explained by the absorption of a stream of Aryan immigrants by the indigenous population.^17^ Similarly, the old philosophical systems were overwhelmed and marginalized by the non-scientific Śaiva and Vaiṣṇava systems. Thus, it is certain, Frauwallner says, that the scientific character of the early systems was racially conditioned. Indian philosophy therefore deserves special attention not only because it represents the most important development in philosophy outside Europe, but also because it is a typical creation of an Aryan nation (“Volk”). Frauwallner approvingly quotes the Orientalist Wolfram von Soden, who claimed that science (Wissenschaft) in the proper sense of the term was something that could be created only by the Indo-Europeans who are determined (“bestimmt”) by the Nordic race. Frauwallner presented the above periodization on two further occasions, once in 1953 in the introduction to the first volume of his *Geschichte der indischen Philosophie*, where the Aryan basis of Indian philosophy is clearly mentioned, albeit less conspicuously (Frauwallner 1953: 26–27),^18^ and, as late as 1959, in his article “Indische Philosophie”, where he claims that classical Indian philosophy in the early period is essentially a creation of the immigrated Aryan Indians.

Frauwallner never renounced his racist theory about the Aryan component in Indian philosophy outlined above. Slaje claims the contrary: “Once the scientific error acknowledged, he no longer advocated the thesis (Slaje 2010: 462).”^19^ To corroborate this claim, he refers the reader to the first volume of Frauwallner’s *Geschichte der indischen Philosophie* published in 1953. Obviously, Slaje forgot what he had read there; he certainly did not bother to read Stuchlik’s book on this point (Stuchlik 2009: 152; see also Steinkellner 2010: xvi and n. 2). Otherwise, even without having read the introduction to *Geschichte der indischen Philosophie* or Frauwallner’s above-mentioned article on “Indische Philosophie”, Slaje would have known that Frauwallner repeated his racist theory not only in 1953, but again in 1959 (Frauwallner 1982a [1959]). Furthermore, as Stuchlik discovered (2009: 187), Frauwallner had not changed his mind even into the late 1960s. Some six years before his death, in a letter to Walter Ruben dated July 17, 1968, which accompanied an off-print of his “Aryan Component in Indian Philosophy”, he writes: “I have indeed found another off-print of my old paper and am sending you the same. You will see that what is essential concerning the distinction of the different orientations in Indian philosophy is already stated there” (quoted in Stuchlik 2009: 187). Nowhere in Frauwallner’s writings do we find a retraction of his theory, and he presumably went on believing it until the end of his life. He also continued to orally express his antisemitic views well into the 1960s.^20^

In 1943, Frauwallner proposed the establishment of a “Department of Orientalistic Indology” within the notorious SS think tank “Deutsches Ahnenerbe” (Stuchlik 2009: 81). The department was to work primarily on the compilation of a new Sanskrit dictionary. In his application, Frauwallner wrote: “However, given the status of German science [Wissenschaft] especially in the field of Indology, it would be a disgrace to rely on foreign dictionaries” (Stuchlik 2009: 82). Had his application been approved, Frauwallner would have become an honorary member of the SS. Fortunately for him, he was spared this disgrace; the war had taken a decisive turn by this point, and on April 30 he was drafted into the army, which prevented him from following up on any further plans regarding the dictionary. One wishes that Stuchlik would have quoted more from Frauwallner’s application.

## After WWII: The Guenther Affair^21^

Herbert Guenther came to Vienna in 1941. He had previously studied Indo-Aryan philology and classics with Walther Wüst and Wilhelm Geiger in Munich, and had written his dissertation on the mixed Prakrit—an earlier term for what later came to be designated as Buddhist Hybrid Sanskrit—of the Mahāvastu, entitled *Grammatik des buddhistischen Mischprakrits. I. Die Sprache des Mahāvastu*. Guenther was extremely gifted with languages, and in addition to Sanskrit, Pali, Sinhalese and Hindi, he knew Tibetan, Chinese and Japanese. In 1943, he obtained his habilitation by submitting a work on Sinhalese grammar, entitled *Das Sidat-saṅgarāva (Zusammenfassung der Regeln): Die einheimische sinhalesische Grammatik. Einleitung, Text und Übersetzung*. However, the conferral of his *venia legendi* was delayed because he had not engaged in any NS activity. Although he declared his willingness to work for the “people’s community” (Volksgemeinschaft), he never joined the Party and does not seem to have been involved in any way with National Socialism. Guenther began teaching at the Department of Oriental Studies of the University of Vienna in October 1943 as a replacement for Frauwallner, who had been drafted earlier that year, and taught continuously as a sessional lecturer until 1948. In 1948, the anthropologist Robert Bleichsteiner, director of Vienna’s Museum of Ethnology, applied for a professorship for Guenther. At that time, Frauwallner, who had been dismissed from the University after the war, was still not permitted to teach. On July 16, 1948, the head of the department wrote to the Ministry of Education that the teaching assignment granted to Guenther by the faculty two weeks earlier had to be withdrawn and announced a disciplinary investigation into Guenther. The application for the professorship fell through, and Guenther eventually lost his employment as a sessional lecturer.

To understand what had happened, one has to go back to 1945. When the war ended in the spring of 1945, there was no trace of Frauwallner; there were rumors that he might have been killed in the war. In May 1945, Frauwallner’s father-in-law transferred Frauwallner’s books (some 400 volumes) to the Department of Oriental Studies; they were deposited in Guenther’s office (Stuchlik 2009: 176). It is not clear under what conditions the books had been deposited; it may be that they were a donation to the department’s library, or considered as such, as Frauwallner later had to receive permission from the Ministry of Education to get the books back. This permission was granted shortly before Frauwallner returned to Vienna in January 1947. While checking his books in Guenther’s office, Frauwallner noticed that some 50 volumes on a certain area of Buddhist studies were missing (Stuchlik 2009: 176). The area is not named in Frauwallner’s report as quoted by Stuchlik, but was probably Early Buddhism. Frauwallner then asked the head of the department, Herbert Duda, to talk to Guenther and request that he return the missing books. Guenther, however, denied any knowledge of them. Frauwallner could not have known this at the time, but this issue was a stroke of luck that would allow him to regain his professorship and save the later part of his career. Had Guenther been appointed, it would have been highly unlikely that the Ministry of Education would have funded a second professorship in Indology, and Frauwallner would have probably remained in early retirement.

Initially nothing came of Frauwallner’s complaint. The books had not been deposited with an accompanying inventory, and it was his word against Guenther’s. Frauwallner, however, began elaborate investigations to prove that Guenther had stolen and used his books. He checked each reference in Guenther’s recent publications (more than 500 pages), made inquiries with Guenther’s students about the texts used in his teaching, sought to find out from various Viennese libraries which books had been borrowed by Guenther over a long period of time, and finally came to a conclusion as to which books Guenther must have had available privately, and to what extent these corresponded to the missing books. He then struck at just the right time. Just as Guenther was being considered for the professorship, Frauwallner approached the head of the department again—this time with evidence—and convinced him that his missing books had indeed been stolen by Guenther. Frauwallner also filed a formal complaint with the police about some 45 books, at an estimated value of 1,000 Reichsmark. In 1949, Guenther was forced to reveal his personal library and returned eight books to Frauwallner. Guenther denied any knowledge of any further books missing from Frauwallner’s collection.

Unfortunately, Guenther’s side of the story is unknown. He may have assumed that Frauwallner had died in the war. That he indeed took some of the books is clear; their actual number could have been anywhere between 8 and 45. When Frauwallner reemerged, Guenther was probably too embarrassed to admit that he had appropriated some of the books and could not simply return them clandestinely because he had erased Frauwallner’s name from the title pages, in order to cover up his misdeed. In any case, Guenther was made to pay a heavy price. He lost his job opportunity at the University and left Austria, moving first to India and then to Canada. His later attempts to establish himself in Austria were successfully blocked by Frauwallner.

## Frauwallner’s Return to the University of Vienna and the South Tyrolean Connection^22^

In connection with Frauwallner’s efforts to pave a way back into academia after his forced retirement, which culminated in the founding of the University’s Department of Indology (Institut für Indologie) in 1955 and his appointment as full professor and head of department, one encounters Dr. Aloys Oberhammer, a member of parliament (Nationalrat) for the conservative Austrian People’s Party (Österreichische Volkspartei, ÖVP) since January 1950, from the federal state of Tyrol. Oberhammer had held various public positions, such as member of the municipal council (Gemeinderat) in Innsbruck (1934–1938) and secretary of the Tyrolean ÖVP (1946–1948); he belonged to the clerical wing of the party (Stuchlik 2009: 153).

Oberhammer and Frauwallner were good friends. Stuchlik found evidence for something that was long rumored, namely that Oberhammer helped Frauwallner gain his professorship (Stuchlik 2009: 153). Frauwallner’s friendship with Oberhammer must have also played a role in the later, unlikely, choice of successor to his Chair, namely, Gerhard Oberhammer, Aloys Oberhammer’s adopted son, who had studied Indology with Frauwallner, but had written his doctoral dissertation at the University of Innsbruck on Plotinus in 1961, and, at the time of his appointment as full professor and head of the Department of Indology in 1964, had only a handful of Indological papers to his credit. As a result, Indology in Austria was to become theologically oriented, with an emphasis on the hermeneutics of religions, as opposed to Frauwallner’s own philological-historical approach, for the next 35 years.

It is not as if there were no other candidates to succeed Frauwallner. Two possible candidates were Guenther and Leopold Fischer, an Austrian who had converted to Hinduism and, as a monk, changed his name to Agehananda Bharati.^23^ If Frauwallner had personal grievances against Guenther, there is no record of anything similar concerning Bharati, a child prodigy who had become Frauwallner’s student at the age of fourteen. Yet in a letter to Dr. Matthias Vereno of the Austrian Society of Religious Studies, dated October 27, 1962, Frauwallner wrote: “To the questionable personalities belongs also [i.e., in addition to Guenther, EF] Mr. Agehananda Bharati, aka Mr. Fischer. Let him stay in America. His return to Austria would yield neither scholarly nor moral gain” (Stuchlik 2009: 169). In this context, as in the Guenther case, Frauwallner’s moral scruples may elicit a smile. How did someone who associated wholeheartedly with a rapacious and murderous pack come to be so deeply concerned about moral gain? The irony of the situation was likely lost on Frauwallner. As pointed out above, he never seems to have felt that there was anything wrong with his earlier association, for more than two decades, with National Socialism. One wonders what moral reproaches he leveled against Bharati. It was certainly not, one can surmise, the latter’s involvement with the Indian Legion. From a scholarly standpoint, Bharati was definitely very talented and turned out to be a prolific and colorful personality in the field of Indology in the twentieth century (on his life, see Bharati 1980), and in spite of his different orientation he would have been a worthy successor to Frauwallner on account of the innovation, creativity, interdisciplinarity and relevance of topics he addressed in his scholarly writings. Bharati’s “sin” seems to have been a critical review of one of Frauwallner’s books, the *Philosophie des Buddhismus*.^24^ He clearly discerned how problematic it would be to see the supposedly “Aryan” Buddhist philosophy as “scientific” and “free from presuppositions”, and to suppress its mystical religious element (Stuchlik 2009: 172). As discussed above, Frauwallner considered the mystical element in Indian philosophy to be the product of the influence of the “non-Aryan” race and foreign to the original, essentially “Aryan” spirit of Buddhism.

## Whitewashing, Downplaying and Denial as Strategies to Come to Terms with Austrian Indology’s National Socialist Past

As should have become clear from the above, Stuchlik has considerably advanced our knowledge about several largely unaddressed aspects of Frauwallner’s life and work. Whatever the faults of his book, it did not deserve to be attacked in the most brutal and contemptuous terms, as Slaje did in his lengthy review, which reflects more on its author than on the book reviewed. Here are some examples of Slaje’s harsh rhetoric against Stuchlik: “Stuchlik’s approach can therefore not be described as anything other than careless” (2010: 450); “contorted verbal acrobatics”; “[Stuchlik has] no method, but an agenda”; “cheap sensationalism/showmanship” (2010: 451); “causal smoke grenades”; “Stuchlik superficially slips into the role of the contemporary historian”; “the story told by Stuchlik about Frauwallner therefore belongs to the ‘story’ category—*nota bene*: of bad ones—, but not to that of history”; “Stuchlik dabbles” (2010: 452); “ludicrous accusations [against Frauwallner]” (2010: 453); “Stuchlik’s method therefore proves to be scheming of the flattest kind” (2010: 456); “the shoddy effort”; “Stuchlik’s twisted causal acrobatics […] in its associative arbitrariness” (2010: 462); “[Stuchlik’s book] has nothing to do with scholarship”; even though it was “inexplicably accepted for publication [by the Austrian Academy of Sciences Press]” (2010: 463).^25^ And so on and so forth. Clearly, Slaje’s furious, abusive and contemptuous response to Stuchlik’s book cannot be understood in merely academic terms, but this is not the place to speculate on the reasons behind such a reaction. To be sure, Stuchlik’s book is not without its problems. One may argue that it overstates its case, is at times unnecessarily speculative and insinuating, or perhaps too one-sided and overly emotional. On the whole, it could have benefited from a rigorous editorial hand. However, with all its shortcomings, Stuchlik’s study remains without any doubt the most significant contribution to date on the history of Indology in Austria during the time of National Socialism. Its painstaking and meticulous research, conducted in numerous archives, is exemplary. Indeed, its greatest merit lies in uncovering significant new material about Frauwallner as a person through a careful reading of unpublished archival documents.^26^ It also sheds some new light on Frauwallner’s “Der arische Anteil an der indischen Philosophie” (1939) by exploring its connection to Hermann Goetz’s *Epochen der indischen Kultur* (Leipzig 1929) and Wolfram von Soden’s article “Leistung und Grenze sumerischer und babylonischer Wissenschaft” (1936).

The rude tone is not the only thing one may object to in Slaje’s review. In his rejoinder, Stuchlik points out that it abounds in tendentious, manipulative misrepresentations of his work. He mentions instances of text manipulation (Textmanipulation), falsification of history (Geschichtsfälschung), lack of attention to detail, and (willful?) misunderstanding. To these points of justified criticism of Slaje’s review, one can add further examples of false arguments, inaccuracies, and so on. My purpose here is, however, not to present a catalog of misgivings. Rather, I want to concentrate on the central point, namely the whitewashing of Frauwallner in terms of his political and moral involvement in National Socialism, racism and antisemitism. Unavoidably, some of my following remarks partly overlap with Stuchlik’s aforementioned response.

What is perhaps most problematic about Slaje’s review is that he seems to think not only that Stuchlik’s book is ill-executed, but that such a study is not even necessary. The period it deals with is a “politically overcome period” (“politisch überwundenen Epoche,” 2010: 452). The implication is clear: what happened during the NS era is a thing of the past, and no longer politically (or otherwise?) relevant. So why bother? Slaje, like Frauwallner, is Austrian, but one wonders whether he has not lost touch with his own country. One could remind him of a few relevant facts that are not part of a bygone past. Austria has until recently has clung to the myth of being the first victim of National Socialism. Wolfgang Schüssel, Austria’s Chancellor from 2000 to 2007, declared in an interview in 2005: “I will never allow anyone not to see Austria as a victim” (Stuchlik 2009: 35, n. 13).^27^ Austria is also a country where the restitution of stolen Jewish property was conducted with much obstruction, hesitation and reluctance (it is still a sad joke); a country whose president clearly lied about his past in WWII; a country where in the early twenty-first century 28% of voters in a national election went for the extreme right-wing party of Jörg Haider, “the best governor of all time”, a man who consistently made antisemitic remarks and publicly said to SS veterans that they were decent (anständig) people and praised the Third Reich for its “appropriate employment policy”; a country where up to 40% of the population still holds typical antisemitic views such as “the Jews have too much power in the world”; a country where by far the most widely read and influential daily, the *Kronenzeitung*, consistently printed antisemitic remarks for more than 50 years; and so on and so forth. It is precisely because Austria failed to unequivocally recognize its role and responsibility in NS atrocities, ignoring, denying or sweeping under the carpet its deep ideological entanglement, that this period has never been “politically overcome”. All of the above was true in 2010 when Slaje wrote his review, and things have not changed much since then. Slaje’s lengthy review is not a bad example of how some Austrians view the past of their country, namely with aggressive denial of its involvement with NS ideology and institutions. On the whole, the review can be seen as a consistent, albeit clumsy and unsuccessful attempt to absolve Frauwallner of any wrongdoing.

In a semblance of a scholarly approach, Slaje acknowledges some facts brought to light by Stuchlik, and then proceeds to strip them of “unbridled chains of association and suggested causal connections”:

As sober facts—beyond unbridled chains of association and suggested causal connections—they present themselves thus: Joining the Austrian NSDAP on November 29, 1932. (p. 35, n. 15)

Membership in the NS Teachers’ Association as of March 14, 1933. (p. 35, n. 15)

Appointment as Extraordinary Professor on August 31, 1939. (p. 64)

Dismissal from public service on June 6, 1945. (p. 84)

Classification as ‘lesser offender’ in 1947. (p. 86)

Confirmation of classification as ‘lesser offender’ on 14 September 1948. (p. 94) (Slaje 2010: 453).

One wonders. Out of all the details uncovered and assembled by Stuchlik, is this all Slaje considers worth mentioning? If he actually read the book he reviewed, one has to conclude that he conveniently suppresses several sobering facts, including Frauwallner’s participation in various other National Socialist and Austro-Fascist organizations, his taking over the flat of his “departed” Jewish neighbors, his failed attempt to acquire it under advantageous conditions, his work for the Party, SA and Gestapo intelligence services, his considerable role in discussions aimed at garnering the support of the Catholic Church for National Socialism, and his (failed) application to the SS think tank “Ahnenerbe.” Are none of these facts sober? Are none worth mentioning in the present context? Are none of these reliable evidence for passing moral judgment?

If one is, like Slaje, blind to all these facts, one may indeed conclude with his incredible sweeping statement that “there is no reliable evidence to pass moral judgment on Frauwallner” (Slaje 2010: 453).^28^ This is arguably the most bizarre statement in the entire review, which is not short of candidates in that category. What does one have to do before one can be morally judged? Is associating formally and factually with a murderous regime not enough? Is never expressing regret, let alone sorrow not reliable evidence? Is being an antisemite morally beyond reproach? How low would Slaje have us set the bar? Ignoring the contradiction, Slaje pleads that, like many others even in prominent public positions, Frauwallner deserved a second chance after the war (Slaje 2010: 462). But only those who are guilty deserve a second chance, and what would Frauwallner be guilty of if there is no reliable evidence for moral judgment? Still, what did Frauwallner do with his second chance? He remained an antisemite, maintained his racist and Aryan-supremacist ideology, and never acknowledged any wrongdoing.

Furthermore, Slaje actually believes, or would have us believe, that whatever happened shortly before and during WWII, as presented by Stuchlik, had nothing to do with Frauwallner (“has no causal connection”): “In tedious length, retellings of long known facts of the NS era or the Second World War are spread out, without these contributing anything to the matter at hand. For they have no causal connection with Frauwallner” (Slaje 2010: 456). Actually, this is not true. Stuchlik’s digressions into descriptions of events before and during WWII are rather short and take up a relatively small part of the book. More than that, it seems that Slaje does not understand why Stuchlik digresses—that is, why he reminds readers of the political context in which Frauwallner’s statements and actions occurred. Apparently Slaje does not comprehend that glorifying the Aryan race in 1938–1939 and 1942–1944, and supporting this glorification with the semblance of a serious scholarly foundation, are not the expression of the mere private opinions of some deluded “public nobody” (Slaje 2010: 462) totally disconnected from the inhumanly cruel political reality in which he is embedded.

## The Defense of Frauwallner’s Hypothesis of the “Aryan Component in Indian Philosophy”

Slaje tries to whitewash not only Frauwallner, but also his notorious paper, “Der arische Anteil an der indischen Philosophie”. As addressed above, Frauwallner divides the history of Indian philosophy into two periods, one characterized by rational philosophy that is “scientific” and “free of presuppositions”, the other theistic and characterized by the powerful religions of Śiva and Viṣṇu. The first is, on the whole, the creation of the Aryan race, the second that of the non-Aryan, that is, indigenous Indian race. Is this not a racist theory clearly positing Aryan supremacy? Not quite, says Slaje. According to him, it is racialist rather than racist. Slaje does not explain his usage of the two terms, but presumably he means that a racialist theory is based on race or racial distinctions, but—contrary to a racist theory—without depreciating one race with respect to another. To substantiate his claim, Slaje points out that when Frauwallner speaks of “Aryan developments”, he does not mean that its “proponents” were throughout (durchwegs) Aryans^29^—“developments” free from any and all foreign influence do not exist anywhere, and especially not in India (2010: 458).^30^ How this is supposed to prove Slaje’s point is unclear. Generally, Frauwallner used the “mixing of races” to explain why Indian philosophy was not quite as “scientific” and “free from presuppositions” as it could have been, had it remained purely Aryan. Specifically, Frauwallner gives the example of Early Buddhism: According to him, its conception of various levels of meditation is related to Laya-Yoga, which is itself related to Tantrism, and is clearly of non-Aryan origin. This is a foreign or alien (fremd) component in Buddhism, which stands in contradiction to its essentially Aryan nature, which is clearly reflected in later Buddhist philosophy. Indeed, Frauwallner that in the *Abhidharmakośa* the various levels or stages of meditation are pushed by Vasubandhu to the background so that they do not constitute a necessary part of the path to liberation, whereas liberating insight is understood as a mere act of intuitive cognition (*abhisamaya*) in accordance with the conceptions (Anschauungen) of Aryan philosophy (1939: 287–288). The racist undertone of these statements is obvious. On top of that, Frauwallner enlists Indology (and perhaps also other disciplines such as Classical Philology) toward the future “observation” of such foreign influences on Aryan philosophy and the determination of their extent. One therefore cannot but ask oneself whether Slaje did actually closely read the paper he defends so fervently or whether he knowingly misrepresented it.

Similarly, one also wonders what led Slaje to state that Frauwallner, unlike his predecessors, did not denigrate the “pre-Aryan exponents” of the second period as a race. Which is puzzling: if not as a race, then as what? Slaje does not say. He enumerates several achievements Frauwallner attributes to the indigenous race, insinuating that Frauwallner did not look down on the “Urbevölkerung” at all. However, this is an apologetic presentation that misrepresents Frauwallner’s clear intention. On one point, however, Slaje is right. Frauwallner at least presents the non-Aryan period “without a touch of emotional prejudice” and “racial hatred”. In this sense, one could say that Frauwallner’s theory is racial rather than racist, but certainly not in the sense implied by Slaje, who adopts it in order to deny Frauwallner’s commitment to Aryan supremacy (a kind of “non-emotional prejudice”?) or belittle it as an innocuous preference of philosophy over theology, which “would be no crime even today (Slaje 2010: 460, see also below)”.

Slaje continues by quoting approvingly two points from earlier essays on Frauwallner by Gerhard Oberhammer (1976: 9 and 1992: 225):

The introduction of the race theory as an inner principle in order to explain these periods is an aberration pertaining to the time, for which Frauwallner had fallen […]. For the ‘Aryan’ spirit that seems to him to characterize the first period of Indian thought is not the spirit ‘of the Aryan race,’ but the spirit of Hellas, which the classical philologist believes he has rediscovered in Ancient India in a related form.

[Frauwallner’s] conception of the periods of Indian philosophy […] is, if one disregards the admittedly untenable thesis of its justification by the racial peculiarity of its representatives, which can only be understood as a fascination, albeit not primarily politically motivated, with the spirit of the time, so far the only scientifically substantiated attempt at a periodization of the history of Indian philosophy derived from typological grounds (2010: 460).

Slaje summarily rephrases Oberhammer’s first point as follows: “From the perspective of his time, his educational horizon and scientific milieu, Frauwallner sought his philosophical ideal of what he himself calls ‘science without presuppositions,’ which he knew from Greece, in India as well”.

First, one may note that Frauwallner does not speak of “science without presuppositions” which would presumably be a pleonasm for him; he rather speaks of philosophy as scientific and without presuppositions, and does not refer to, or deal with, science per se. Further, he did not “know” (or “discover”, as is implied by Oberhammer) his philosophical ideal (or the “spirit of Hellas”, as Oberhammer put it) from Ancient Greece, but projected it both onto Greek Antiquity and—with some discrimination—Ancient India. But that being said, does Frauwallner’s supposed confusion of the ancient Greeks with the Aryans (in the sense of Indo-Europeans) make him less of a racist? Were the ancient Greeks not also considered Aryans? Why does Frauwallner’s projection of an ostensible Greek ideal onto Aryan India make him less of a racist? If I were wrongly to believe that, like all Jews, Indian Jews have crooked noses and are financial wizards, and it turned out that this was based solely on my mistaken projection of certain characteristics onto the Greek Jewish community, would I then be less of an antisemite? One may add, in connection, that Frauwallner not only subscribed to the supremacy of the Aryan race, but also, like von Soden and many others, believed in the superiority of the Germanic or Nordic branch thereof.

## Can Frauwallner’s Periodization of Indian Philosophy, Divorced from its Racist or Racialist Foundation, be Defended?

Concerning the second point made by Oberhammer, he and Slaje would have us believe that if one only separated Frauwallner’s theory from its racial foundation, which is supposedly due to some unfortunate misunderstanding and infatuation with a dominant contemporary ideology, it would emerge as a ground-breaking valuable scholarly theory about the periods of Indian philosophy, rooted in true scholarship and derived from typological grounds. As I have pointed out elsewhere,^31^ even if one disregards the questions of whether it is fitting to divide the entire history of Indian philosophy into two periods, and whether there was philosophy at all in India in the eighth and subsequent centuries BCE,^32^ it is unclear in what sense the Buddhist and Jain philosophies can be said to be non-religious. Further, there is a considerable time gap between the arising of the supposedly non-Aryan religions of Viṣṇu and Kṛṣṇa and the emergence of the supposedly non-Aryan philosophies. The former are already emerging in the *Mahābhārata*; yet the philosophy of the Epic represents, even according to Frauwallner, a period which precedes the formation of the classical, presumably Aryan philosophical systems of Sāṃkhya and Vaiśeṣika (see Frauwallner 1984, see also criticisms by Bakker & Bisschop 1999 and Preisendanz 1994).^33^ Further, the terms “scientific”, “without presuppositions”, “atheistic”, and so forth, in their usual meaning can hardly be accepted to describe Indian philosophy in the early and classical period (or any other period), and it is only with the peculiar, very narrow and somewhat arbitrary meaning ascribed by Frauwallner that they could be deemed apposite for a description of Indian philosophy or any other philosophy. In Frauwallner’s sense of atheism, both Vedic and Greek religion could be said to be “atheistic”.

However, the most intriguing part of Frauwallner’s periodization is that he regards the second half of the first millennium CE, the period on which he has done much of his best work, to be a period of decline. It is difficult to see how one could claim that the scientific character of Indian philosophy that in Frauwallner’s own words is manifest in epistemology and logic forming the basis of every proof^34^ could be said to reach its peak in the first, rather than the second half of the first millennium. It also seems odd that the period of the brightest stars in Indian philosophy—Dignāga, Praśastapāda, Uddyotakara, Prabhākara, Kumārila, Dharmakīrti, Akalaṅka, Dharmottara, Maṇḍanamiśra, Jayarāśi, Prajñākaragupta, Jayanta, Bhāsarvajña, Udayana, and so forth—should be considered a period of decline, and labeled somewhat derogatively as “the period of logico-epistemological speculation” (Frauwallner 1953: 25).

Therefore, the opposite conclusion suggests itself, namely, that Frauwallner’s periodization has little or nothing to do with typological features of Indian philosophy and much—or everything—to do with the presumed racial division into Aryan and non-Aryan. In other words, if we were to apply Frauwallner’s own criterion of being “scientific” or “without presuppositions” to the history of Indian philosophy, we would have to put his theory on its head inasmuch as the development of various theories of means of knowledge (*pramāṇa*) and their application to the exploration of major philosophical topics between the fifth and eleventh centuries, and the flourishing of Navya Nyāya from the eleventh century onward, would make the so-called non-Aryan period much more “scientific” than the Aryan one. Frauwallner was neither original nor exceptional in his assessment of Indian civilization, an assessment strongly influenced by the consideration of “race”. He directly refers to the following passage by the great Vedic and Buddhist scholar Hermann Oldenberg, whom he admired and regarded as brilliant, and who seems to have been a source of direct inspiration to him:

Above all there were probably influences [by the indigenous people of India] that worked in a very profound way which we can only surmise: through the gradually progressing transformation of the blood, which means a transformation of the Soul, through the constant influx of new quantities of the blood of savages and semi-savages into the veins of those who still called themselves Aryans. Zeus and Apollo continued to rule as long as there were Greek gods because the Greek nation remained the same. Indra and Agni had to leave the field to other gods because the Indian nation had become a different one. For these minds, in which an inscrutable jumble of antagonistic powers, intertwined with each other, unleashed at each other, was at work, the Vedic gods were much too guilelessly simple; their being was all too easily exhausted. They had come from the North: now tropical gods were needed. These were hardly of fixed shapes any longer; they were whole tangles of shapes, bodies from which oozed heads upon heads, arms upon arms, multitudes of hands holding multitudes of attributes, clubs and lotus flowers: voluptuous, somber and grandiose poetry everywhere, exuberance and blurred shapelessness: a terrible disaster for the fine arts (Oldenberg 1923: 132–133).

## The Representation of Frauwallner As a Lover of Philosophy and Brave Dissident During the Years of WWII

Frauwallner’s direct reference to Oldenberg’s work as a source of inspiration does not prevent Slaje from rephrasing the distinction made by Frauwallner between the races so gently, as if it were only a matter of legitimate sympathy for supposedly pure philosophy which is devoid of any dogmata, as opposed to theology which is endowed with presuppositions and fraught with dogmata: “So if [note the conditional; EF] Frauwallner did take a judgmental stance, it was located in the field of tension between theology and philosophy, as a legacy of European intellectual history. Here he no doubt sympathized with philosophy because it was devoid of presuppositions and free of dogmata. That would be no crime even today” (Slaje 2010: 460).

So this is what Slaje would have us believe. Frauwallner was no racist, only a misguided racialist who “succumbed” (Oberhammer’s term) to an error prevalent in his time and who merely had more sympathy for philosophy than for theology. Occasionally, the obnoxious becomes comical. Slaje would also have us believe that Frauwallner was actually a brave dissident: “On the contrary, Frauwallner had the greatness and courage to explicitly and publicly acknowledge the cultural achievements of India’s ‘non-Aryan indigenous population,’ even in the war year 1942 in Berlin, in a lecture entitled ‘The Significance of Indian Philosophy,’ which was published in 1944” (2010: 460–461).

## Where are the German Indologists?

Slaje complains that Stuchlik lacks the qualifications of a modern historian. Stuchlik, however, took the trouble to consult modern historians when writing his study. It would have been wise for Slaje to have done the same before the publication of his review. As stated above, the fact that Slaje’s review amounts to an implausible attempt to whitewash Frauwallner and the history of Austrian Indology during the NS period does not mean that Stuchlik’s study is perfect or beyond critique. The nagging question is rather why nothing better, more comprehensive, and based on interdisciplinary theoretical considerations, has thus far been written on the subject. Why is it that much research has been done by Indologists and historians alike on the history of Indology in the German-speaking world before the NS period, but that the task of coming to terms with the discipline’s NS past had to be taken up, as late as the 1990s, by an American Indologist like Pollock, followed by German scholars of religion like Junginger, a Polish inter-cultural philosopher like Stuchlik, and a historian of modern South Asia like Framke? Where were the German Indologists, now all deceased, who experienced the discipline—as students and scholars—during the NS time? One can only hope that the gloomy picture of aggressive denial conveyed by Slaje (and before him by Grünendahl) is not representative of the attitude of present-day German Indologists as a whole; even so, all others, one notes sadly, have remained hitherto silent.

## Footnotes

1 See also Houben (1994). On Bernhard Breloer, Ludwig Alsdorf and Devendra Nath Bannerjea, see Framke (2014); on Johannes Hertel, see Neubert (2012); on Heinrich Zimmer, see Roy (2022).

2 As an exception, one may mention Hanneder (2010) on Johannes Nobel. However, this study hardly counts. Hanneder only summarizes Nobel’s personnel file from the Marburg University archives; the central piece of information that we gain from this study is that Nobel liked to travel and wanted to spend a four week holiday in Italy.

3 Such objections have not come just from Indologists. I will not discuss here the attempt by Rüdiger Schmitt to whitewash Frauwallner’s racist interpretation of the history of Indian philosophy, as treated by Stuchlik, by dwelling on different meanings of the term “Aryan” and keeping completely silent on Frauwallner’s involvement with National Socialist institutions (Schmitt 2010/2011). I will also not discuss any short book reviews here, such as those by Jürgen Hanneder and K.T. Schmidt.

4 Stuchlik defended his study in a rejoinder, where he demonstrated inter alia the manipulations and distortions of Slaje’s review (Stuchlik 2011).

5 As can be expected, this controversy is almost completely unknown outside the narrow circle of German Indologists. What however surprised me, and also motivated me to write the present article, is that I discovered that the controversy has remained under the radar even of some German Indologists.

6 “Eine belastbare Beleglage für eine moralische Verurteilung Frauwallners fehlt.”

7 All details of Frauwallner’s life and academic career in Vienna presented in the following paragraphs are taken from various documents preserved in the archives of the University of Vienna, as collected by Karin Preisendanz. Some of these details, with precise archival references, are also pointed out by Stuchlik in his study. See also Franco and Preisendanz (2010: XVIIIff).

8 “als eifriger Besucher nationalsozialistischer Versammlungen, […] und hat dem Vernehmen nach auch selbst in einer solchen Versammlung gesprochen”. The police report is dated 28.06.1935.

9 On the use of the term “Stellenraub” in this context, see Stuchlik (2009: 150, n. 679).

10 Is this a mistake for April 22? See Stuchlik (2009: 63).

11 See https://gedenkbuch.univie.ac.at/index.php?id=429.

12 In this section, I summarize Stuchlik’s research (2009: 130ff).

13 Stuchlik (2009: 133, n. 700): “Ich habe bisher kein Judenvermögen erworben”.

14 The following section is adapted from Franco & Preisendanz (2010) with some additions. The presentation overlaps only partly with Stuchlik’s extensive deliberations.

15 Frauwallner (1939: 271): “nicht religiös-dogmatisch gebunden, [...] suchen ihre Lehren wissenschaftlich voraussetzungslos abzuleiten”.

16 Frauwallner (1939: 285): “daß es sich um einen Sieg nichtarischen Wesens über die ermattende Kraft des in den älteren Systemen schöpferischen arischen Geistes handelt”.

17 Frauwallner (1939: 285): “mit der Aufsaugung des arischen Einwandererstromes durch die Urbevölkerung begründet”.

18 Note Frauwallner’s usage of the typical NS term “Volkskörper” (1953: 26).

19 On Slaje’s attempts to defend Frauwallner’s hypothesis, see below.

20 This is known from personal communications with colleagues who knew Frauwallner in the 1960s; see also Stuchlik’s rejoinder (2011: 297) where he quotes a personal communication from Lambert Schmithausen that Frauwallner did not break with his past (“mit seiner Vergangenheit nicht gebrochen hatte”). In a further personal communication, Professor Schmithausen told me what he meant, namely, that to his dismay he discovered that as late as the 1960s Frauwallner still believed that WWII was unleashed by Poland’s attack on Germany.

21 For this section, see Stuchlik (2009: 173–186).

22 For this section, see Stuchlik (2009: 153ff).

23 The final list had only two names on it, Oberhammer and Paul Thieme (Stuchlik 2009: 64, n. 240).

24 See Bharati (1960). Parts of this review are quoted in Stuchlik (2009: 172).

25 “Das Vorgehen Stuchliks kann man daher nicht anders als hemdsärmelig bezeichnen” (Slaje 2010: 450); “krause Wortakrobatik”; “[Stuchlik hat] keine Methode, aber Absichten”; “Effekthascherei” (Slaje 2010: 451); “kausale Nebelgranaten”; “Stuchlik schlüpft […] äußerlich in die Rolle des Zeithistorikers”; “Die von Stuchlik erzählte Geschichte über Frauwallner gehört all dem zufolge in die Kategorie der Geschichten—*nota bene*: der schlechten—, aber nicht der Geschichte”; “Stuchlik dilettiert” (Slaje 2010: 452); “aberwitzige Schuldzuweisungen” (Slaje 2010: 453); “Stuchliks Methode erweist sich damit als Berechnung plattester Art” (Slaje 2010: 456); “das Machwerk”; “Stuchliks verdrehte Kausalakrobatik […] in ihrer assoziativen Beliebigkeit” (Slaje 2010: 462); “Mit Wissenschaft hat es [Stuchliks Buch] nichts zu tun” (Slaje 2010: 463); “[im Verlag der Österreichischen Akademie der Wissenschaften] unerklärlicherweise erscheinen durfte” (Slaje 2010: 463).

26 For a list of archives consulted, see Stuchlik (2009: 29–31).

27 “Ich werde nie zulassen, dass man Österreich nicht als Opfer sieht”.

28 For the original, see endnote 7.

29 “Frauwallner stellt dabei aber deutlich heraus, daß, wenn er eine Entwicklung als ‘arisch’ bezeichne, damit natürlich nicht gemeint ist, daß ihre Träger durchwegs Arier waren. Vor diesem Hintergrund ist Frauwallners Periodisierung als ‘rassisch’, nicht—wie von Stuchlik unterstellt (S. 9)—als ‘rassistisch’ zu sehen: ‘[…] die erste [Entwicklungsperiode ist] ihrem Wesen nach arisch, als ihre Träger sind die eingewanderten arischen Inder zu betrachten. Die zweite ist unarisch, sie ist eine Schöpfung der vorarischen Bevölkerung Indiens (Slaje 2010: 458).’”

30 Slaje (2010: 458), quoting Frauwallner (1939: 287): “Eine solche von jedem fremden Einfluß freie Entwicklung” fände sich “überhaupt nirgends, am wenigsten in Indien.”

31 On the periodization and historiography of Indian philosophy, see Franco (2013: 9–11). The following is a quotation with minor changes and additions.

32 I cannot enter into the question here when philosophy begins in India, which depends on the definition of philosophy one has in mind. However, if one adopts Frauwallner’s own notion of philosophy as a “scientific” endeavor, then, ironically enough, philosophy in India in the proper sense begins only in Frauwallner’s non-Aryan period or at best in his “period of decline” of the Aryan period in approximately the fifth century CE. Moreover, contrary to Oberhammer’s claim, Frauwallner’s was not the first and “to date only” attempt to suggest a “scholarly” periodization of Indian philosophy. On Paul Deussen, Walter Ruben and Madeleine Biardeau’s periodizations, see Franco (2013). That essay also contains an appendix by Shinya Moriyama on periodizations of Indian philosophy by Japanese scholars.

33 Independently of its racist or racialist basis, Frauwallner’s reconstruction of the early history of Sāṃkhya has been completely discredited by Motegi (2013). Even earlier, Hans Bakker and Peter Bisschop criticized Frauwallner’s reconstruction of early Sāṃkhya. His reconstruction of the history of Vaiśeṣika has been shown to rest on shaky foundations by Preisendanz (1994). In view of these and other studies, it has become clear that Frauwallner’s methodology relies too heavily on his own imagination and not infrequently arbitrarily uses textual evidence to make it fit.

34 See above, p. ##.
